# The SDS22:PP1:I3 complex: SDS22 binding to PP1 loosens the active site metal to prime metal exchange

**DOI:** 10.1016/j.jbc.2023.105515

**Published:** 2023-11-30

**Authors:** Meng S. Choy, Gautam Srivastava, Lucy C. Robinson, Kelly Tatchell, Rebecca Page, Wolfgang Peti

**Affiliations:** 1 Department of Molecular Biology and Biophysics, University of Connecticut Health Center, Farmington, Connecticut, USA; 2 Department of Biochemistry and Molecular Biology, Louisiana State University Health Sciences Center, Shreveport, Louisiana, USA; 3 Department of Cell Biology, University of Connecticut Health Center, Farmington, Connecticut, USA

**Keywords:** biogenesis, dynamic interaction, enzyme inhibition, inhibitor-3, metal binding, metal loading, NMR, protein phosphatase 1, SDS22, Ser/Thr protein phosphatase, SPR, X-ray crystallography

## Abstract

SDS22 and Inhibitor-3 (I3) are two ancient regulators of protein phosphatase 1 (PP1) that regulate multiple essential biological processes. Both SDS22 and I3 form stable dimeric complexes with PP1; however, and atypically for PP1 regulators, they also form a triple complex, where both proteins bind to PP1 simultaneously (SPI complex). Here we report the crystal structure of the SPI complex. While both regulators bind PP1 in conformations identical to those observed in their individual PP1 complexes, PP1 adopts the SDS22-bound conformation, which lacks its M1 metal. Unexpectedly, surface plasmon resonance (SPR) revealed that the affinity of I3 for the SDS22:PP1 complex is ∼10-fold lower than PP1 alone. We show that this change in binding affinity is solely due to the interaction of I3 with the PP1 active site, specifically PP1’s M2 metal, demonstrating that SDS22 likely allows for PP1 M2 metal exchange and thus PP1 biogenesis.

The ser/thr protein phosphatase (PPP) family is essential for most dephosphorylation events in higher eukaryotes (1). Protein Phosphatase 1 (PP1; 37.5 kDa) is the most widely expressed and abundant PPP (2). Dephosphorylation events catalyzed by PP1 regulate hundreds of processes, including cell-cycle progression, protein synthesis, muscle contraction, carbohydrate metabolism, transcription, and neuronal signaling, among others. PP1 requires two metal ions at its active site for folding and activity (M1 and M2; coordinated by three and four residues, respectively), as they activate a water molecule to facilitate phosphate hydrolysis from substrates (3, 4, 5). Expressing PP1 in bacteria (*Escherichia coli*) requires the media to be supplemented with MnCl_2_ to facilitate PP1 folding; the metals bound to PP1, M1, and M2, are thus Mn^2+^ (other metals can also be supplemented during PP1 expression, including Zn^2+^, Fe^2+^, Ni^2+,^ and Cu^2+^ (5, 6)). In contrast, expressing PP1 in mammalian Expi293 cells does not require the addition of exogenous metals during expression. X-ray fluorescence spectroscopy experiments of crystals containing mammalian purified PP1 always detect Zn^2+^ in M1. However, our data show that M2 can vary and is either Fe^2+^ or Zn^2+^; this likely depends on the availability of Fe^2+^. The metal biogenesis of PPPs, especially PP1, is currently poorly understood.

PP1 interacts with >200 known regulatory proteins, which target PP1 to distinct cellular compartments and direct its substrate specificity (7, 8). The majority of these regulators are intrinsically disordered proteins (IDPs) and bind PP1 *via* an RVxF short linear motif (SLiM) (9); however, additional motifs beyond the RVxF SLiM are often critical for cellular function and allow for a molecular distinction between these regulators (1, 10). A major question in the field is the mechanism by which these regulators both assemble with PP1 and exchange to form new PP1 holoenzymes. Two of the most evolutionarily ancient regulators of PP1 that participate in these processes are suppressor-of-Dis2-number 2 (SDS22; PPP1R7) (11) and Inhibtor-3 (126 aa; I3; PPP1R11 also referred to as YPI1, HCGV, IPP3, or TCTEX5) (12, 13). Both genes are essential in yeast, as their deletion is lethal (14, 15), often resulting in mitotic arrest phenotypes (16).

As is typical for most PP1 regulators, SDS22 and I3 form two distinct heterodimeric complexes with PP1 (SDS22:PP1 and I3:PP1) and, when bound, each potently inhibits PP1 activity (14, 17, 18, 19) (Fig. 1). SDS22, a folded leucine-rich repeat protein (one of the few regulators that does not have an RVxF motif) (11, 20, 21), was recently shown to exclusively bind PP1 that lacks its M1 metal (M1 metal-deficient PP1) (22). Because both metals are essential for PP1 activity, SDS22-bound M1 metal-deficient PP1 is inactive. It was also shown that in the absence of external factors, this binding is constitutive (22). These data are consistent with a model in which SDS22 serves as a cellular PP1 ‘storage’ protein, maintaining PP1 in an inactive state until needed for holoenzyme formation with other PP1-specific regulators (22, 23). Unlike SDS22, I3 is an IDP that contains a canonical PP1-specific RVxF SLiM (12, 14). Structural and biophysical studies subsequently showed that I3 binds PP1 using not one, but two canonical PP1 SLiMs, the RVxF and SILK SLiMs (24, 25). However, they also revealed that I3 uses a novel dynamic (fuzzy) interaction mediated by a three cysteine residue (CCC) motif to engage the active site M2 metal and inhibit PP1 (24). Further studies of this dynamic interaction established that only one of the three cysteine residues is necessary for the binding and inhibition to occur (24). As is typical for such dynamic interactions, only very limited density at this site is observed in the crystal structure, and instead orthogonal approaches, including NMR spectroscopy, mutagenesis, and SPR, were needed to confirm and understand this interaction.

**Figure 1:**
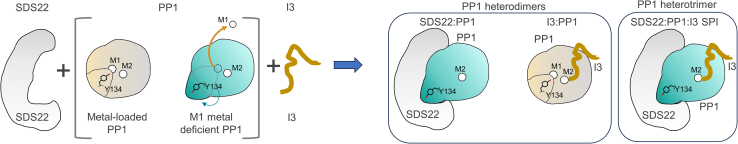
**Complexes formed between SDS22, Inhibitor-3 (I3) and PP1.** SDS22 (*grey*), I3 (*yellow*) and PP1 (which, in solution, exchanges between the M1 metal-bound [beige] and M1 metal-deficient [*teal*] states) associate to form either two distinct heterodimers (SDS22:PP1; I3:PP1) or a heterotrimer (SDS22:PP1:I3, SPI).

Atypically for PP1, SDS22 and I3 also form a heterotrimeric complex with PP1 (SDS22:PP1:I3; hereafter referred to as SPI) (26, 27) (Fig. 1). Work during the last decade has shown that the SPI complex, *via* I3, is specifically recruited to the AAA+-ATPase p97 (VCP or Cdc48) in a ubiquitin-independent manner (28, 29, 30). Once recruited, the SPI complex is disassembled, allowing PP1 to assemble with PP1-specific regulators to form distinct, active holoenzymes (28, 31). How this assembly is achieved at a molecular level is a process that is only beginning to be understood.

One key step to answer this question is to determine if and how the SPI complex differs from the individual SDS22:PP1 and I3:PP1 dimeric complexes. Overlaying the structures of the SDS22:PP1 and I3:PP1 complexes shows that the interaction surfaces of SDS22 and I3 on PP1 do not overlap (22, 24), consistent with their ability to form a trimeric complex. However, the conformations of PP1 in the SDS22:PP1 and I3:PP1 complexes differ. Specifically, in the SDS22:PP1 complex, the loss of the PP1 M1 metal allows the Tyr134 helix to unwind, moving away from the active site to bind directly to SDS22 (22). In contrast, the structure of the I3:PP1 complex contains both metals at the PP1 active site, with PP1 Tyr134 in the M1-metal-bound formation (24). The conformation adopted by PP1 in the SPI complex remains an open question. Here we report the crystal structure of the SDS22:PP1:I3 (SPI) complex, expressed in mammalian cells. The structure shows that both regulatory proteins bind as expected from their individual PP1 complexes. Despite this, our biophysical data show that the binding strength of I3 has changed. Namely, surface plasmon resonance spectroscopy (SPR) showed that I3 binding to SDS22:PP1 is ≥10-fold weaker than binding to PP1 alone. Furthermore, we show that this is due to a change in its interaction with the PP1 active site and highlights a previously unidentified role of SDS22 to weaken the affinity of the M2 metal, likely facilitating metal exchange and/or metal insertion. This further emphasizes the critical role of SDS22 in the biogenesis of metal-loaded PP1 (23).

## Results

### The SDS22:PP1:I3 (SPI) complex

**Figure 2:**
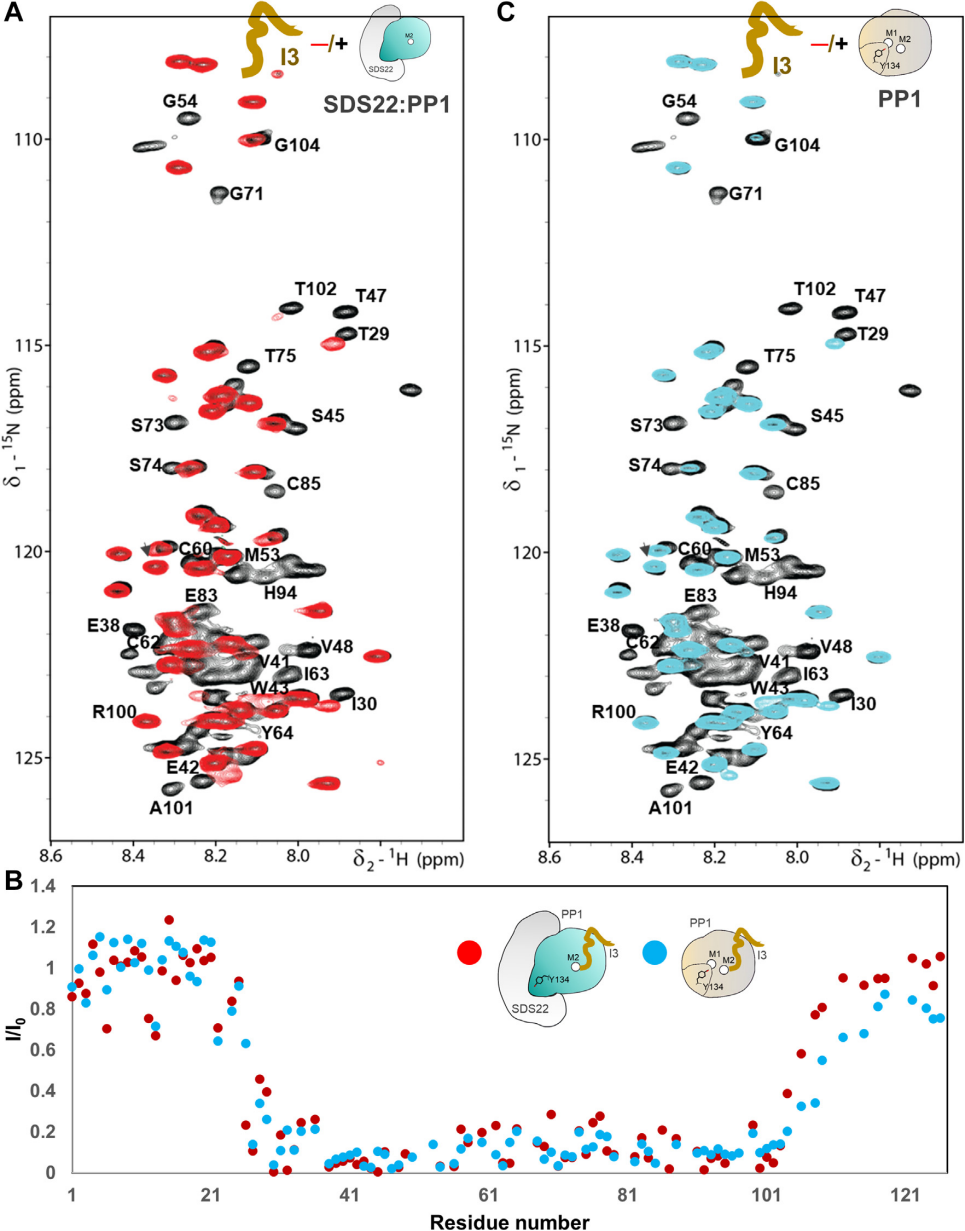
**I3 interaction with SDS22:PP1.***A*, 2D [^1^H,^15^N] HSQC spectrum of ^15^N-labeled I3_1-126_ alone (*black*) and in complex with SDS22_56-360_:PP1α_1-330_ (*red*). *B*, peak intensity loss of I3 *versus* I3 protein sequence plot; SDS22:PP1 (*red*); PP1 (*blue*). *C*, 2D [^1^H,^15^N] HSQC spectrum of ^15^N-labeled I3_1-126_ alone (*black*) and in complex with PP1α_1-330_ (*teal*).

Inhibitor-3 (I3) is a small 126 aa intrinsically disordered protein (IDP) without preferred secondary or tertiary structure (24, 25). To identify how I3 binds to the SDS22:PP1 complex, we recorded the 2D [^1^H,^15^N] HSQC spectra of free and SDS22:PP1α_1-330_-bound ^15^N-labeled I3 (Fig. 2*A*). In this experiment, H^N^/N cross-peaks corresponding to I3 residues that bind SDS22:PP1 disappear due to the high molecular weight of the complex (∼100 kDa) (Fig. 2*B*), with the remaining visible I3 H^N^/N cross-peaks corresponding to residues that do not bind (32, 33, 34, 35). H^N^/N cross-peaks corresponding to I3 residues ∼25 to 100 disappear in the presence of SDS22:PP1, demonstrating that I3 interacts extensively with SDS22:PP1 (Fig. 2, *A* and *B*).

**Figure 3:**
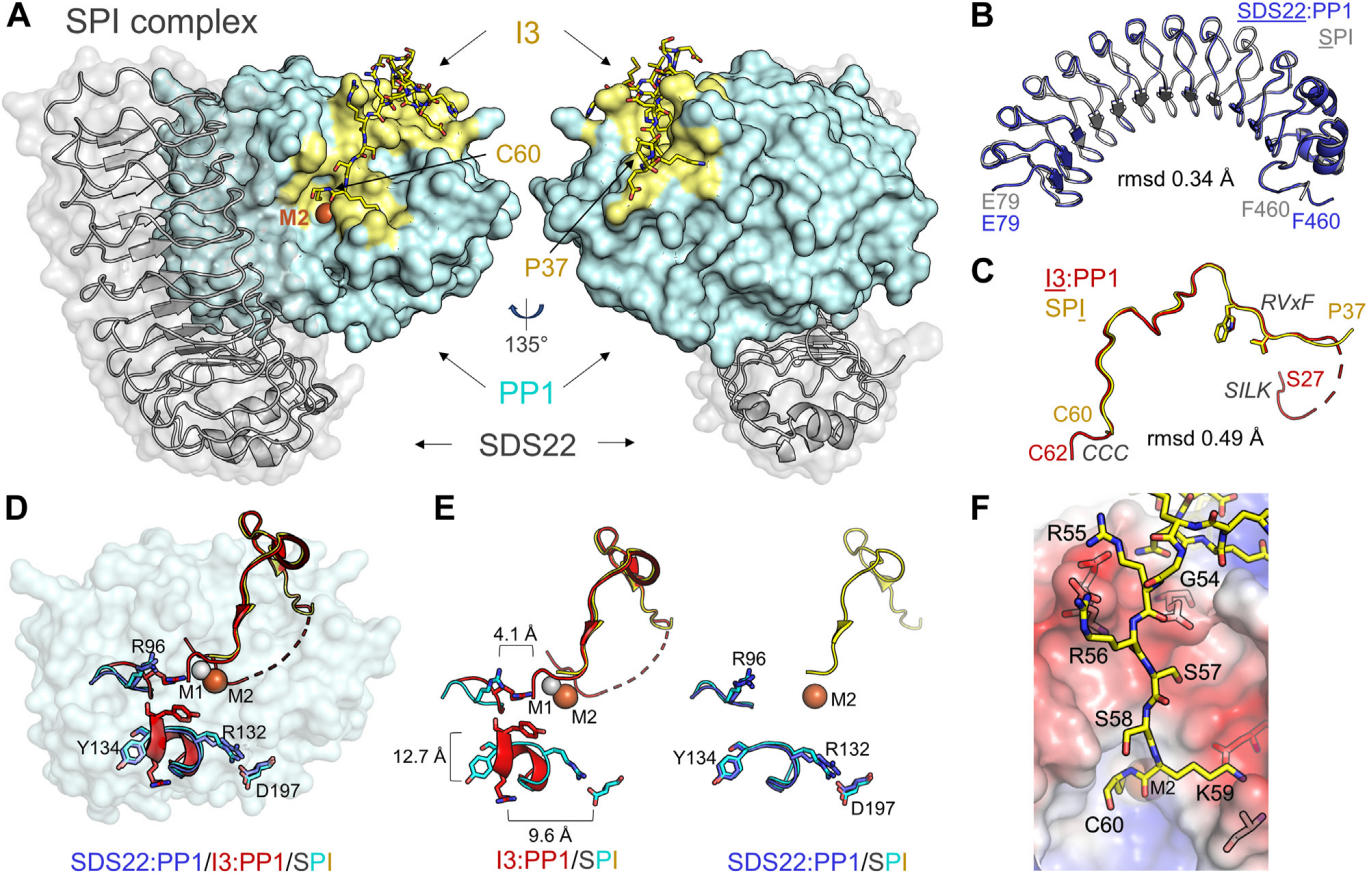
**The SPI holoenzyme complex.***A*, the structure of the SPI complex (SDS22: *grey* transparent surface shown as a cartoon; PP1, *light cyan* surface; M2 metal shown in *orange*; I3: *yellow sticks*; PDBid: 8U5G). The I3-interaction surface on PP1 is highlighted in *yellow*. The N- and C-terminal residues of I3 are labeled (Pro37 [P37], Cys60 [C60]). *B*, overlay of SDS22 from the SPI (*light grey*) and SDS22:PP1 (*lavender*; PDBid: 6OBN)) complexes (rmsd, 0.34 Å). *C*, overlay of I3 from the SPI (*yellow*) and I3:PP1 (*red*; PDBid: 8DWL) complexes (rmsd, 0.49 Å). *D*, overlay of PP1 (and I3, when present) from the SDS22:PP1 (*lavender*), I3:PP1 (*red*) and SPI (*cyan*, *yellow*) complexes; I3 shown as a ribbon and the PP1 Y134 loop, R96 and D197 residues shown as sticks. *E*, same as (*D*) but with only I3:PP1 and SPI (*left*; distance changes in PP1 residue conformations due to M1-metal loss and stabilization by SDS22 are indicated) or SDS22:PP1 and SPI (*right*; key PP1 residues labeled). *F*. interaction of I3 (*yellow sticks*) and PP1 (shown as an electrostatic surface) in the SPI complex at the PP1 acidic groove and active site (identified by the presence of the single bound M2 metal).

It is well-established that I3 binds to both PP1 alone (I3:PP1, dimer) and the SDS22:PP1 complex (SDS22:PP1:I3, SPI, trimer) (12, 27). To determine if the interaction of I3 is similar (or different) in the trimeric and dimeric complexes, we compared the overlays of the 2D [^1^H,^15^N] HSQC spectra of ^15^N-labeled I3 bound to either SDS22:PP1 (Fig. 2*A*) or PP1 (Fig. 2*C*; see also (24)). The bound spectra of ^15^N-labeled I3 in both complexes are nearly identical (Fig. 2, *A* and *C*). Together, these data suggest that the interaction of I3 with SDS22:PP1 is highly similar to that observed with PP1 alone and does not involve additional interactions with SDS22. To confirm this, we used X-ray crystallography. The SPI complex (SDS22_56-360_, PP1α_7-300_ and I3_27-68_) was co-expressed in Expi293F cells and purified to homogeneity (Fig. S1*A*). After extensive efforts, diffraction-quality crystals were obtained (Fig. S1, *B* and *C*), and the structure was determined to a resolution of 3.2 Å. Interpretable electron density was observed for SDS22 residues 79 to 360, PP1 residues 7 to 300, and I3 residues 37 to 60 (Fig. 3*A*; see Table 1 for data collection and refinement statistics) and a single metal, M2, at the PP1 active site. Previously, we determined the structures of SDS22:PP1 (22) and I3:PP1 (24). Overlaying the structures of SDS22 from the SDS22:PP1 and the SPI complexes showed that SDS22 does not change conformation when I3 is bound (root mean square deviation, RMSD, 0.34 Å; Fig. 3*B*). Similarly, overlaying the structures of I3 from the I3:PP1 and SPI complexes showed that the conformation of I3 is largely similar in the presence and absence of SDS22 when bound to PP1 (RMSD, 0.49 Å, Fig. 3*C*). We previously showed that PP1 changes conformation when bound to SDS22, with the Tyr134 helix unwinding to allow Tyr134 to bind directly to SDS22 and simultaneously lacking the M1 metal (22). Overlaying the structures of PP1 from the SPI and either the I3:PP1 (RMSD, 0.97 Å) or SDS22:PP1 (RMSD, 0.47 Å) complexes showed that PP1 adopts the SDS22-bound conformation in the SPI complex, in which PP1 residue Tyr134 unwinds to bind SDS22. Consistent with this, only the M2 metal is present at the PP1 active site of SPI (Figs. 3, *D* and *E* and S2).

**Table 1:** Data collection and refinement statistics

	SDS22_56-360_:PP1α_7-300_:I3_27-68_^a^
PDBID	8U5G
Beamline	SSRL 12–2
Wavelength (Å)	0.97946
Data collection	
Space group	F 4 3 2
Cell dimensions	
*a*, *b*, *c* (Å)	334.80, 334.80, 334.80
α, β, γ (°)	90.00, 90.00, 90.00
Resolution (Å)	3.20
*R*_merge_	0.48 (3.40)
*I*/σ*I*	7.4 (1.1)
CC half	0.80 (0.50)
Completeness (%)	99.9 (100.0)
Redundancy	19.6 (20.1)
Refinement	
Resolution (Å)	39.46–3.20 (3.24–3.20)
No. reflections	27,014
*R*_work_/*R*_free_	0.21 (0.38)/0.24 (0.41)
No. atoms	
Protein	4807
Ligand/ion	44
Water	32
*B*-factors	
Protein	70.07
Ligand/ion	104.53
Water	46.31
R.m.s. deviations	
Bond lengths (Å)	0.002
Bond angles (°)	0.479
Ramachandran	
Outliers (%)	0.00
Allowed (%)	7.39
Favored (%)	92.61
Rotamer Outliers	0.56
Clashscore	4.02

∗Values in parentheses are for highest-resolution shell.

a Data was collected from a single crystal.

### I3 binding to the SDS22:PP1 complex

The interaction between I3 and SDS22:PP1 buries nearly 2500 Å^2^ of solvent-accessible surface area (Fig. 3*A*). Interpretable electron density was observed for I3 residues 39 to 60; this differs from the I3:PP1 complex, in which well-ordered electron density was observed for I3 residues 27 to 31 (SILK motif, ^28^LITK^31^) and 38 to 62 (RVxF, ^40^RVEW^43^ and CCC, ^60^CCC^62^) (Fig. 3*C*). Although we showed that the I3 SILK motif binds PP1 in solution and in crystalloid, we also showed that this interaction does not significantly contribute to the PP1 binding affinity (24). Consistent with this observation, others have also shown that residues immediately N-terminal to the I3 SILK motif facilitate the recruitment of the AAA+-ATPase p97 to the SPI complex (31), demonstrating these residues must be accessible in the SPI complex. Together, these data demonstrate that the I3 SILK motif is dispensable for I3-mediated recruitment of the SDS22:PP1 complex.

The remainder of the I3 interaction with SDS22:PP1 in the SPI complex mimics that observed in the I3:PP1 complex. First, the I3 residues that connect the SILK and RVxF binding motifs (^32^LRKRKP^37^) lack electron density (Fig. S3); *i.e.*, these residues remain dynamic when I3 binds PP1 and the SDS22:PP1 complex. These residues function as the I3 nuclear localization signal, NLS, as a major function of I3 is to translocate PP1 to the nucleus (36, 37). Second, residues 39 to 60 bind essentially identically between the two structures (I3:PP1 and SPI complexes) (Fig. 3*C*). These residues constitute the RVxF SLiM, the acidic groove interaction sequence, and the start of the CCC motif. I3 binding is stabilized *via* an extensive network of polar and hydrophobic interactions. In particular, I3 residues ^54^GRRSS^58^, which bind the PP1 acidic substrate binding groove, are stabilized by electrostatic interactions (Arg55_I3_ and Arg56_I3_ binding Glu252_PP1_, Asp253_PP1_, Glu275_PP1,_ and Glu256_PP1_) (Fig. 3*F*). Compared to the electron density in the I3:PP1 complex, the electron density for these interactions is less defined in the SPI complex, indicating that they may be more dynamic in the SPI complex. However, as observed in the I3:PP1 structure, Lys59_I3_ is well-ordered, binding a deep, acidic pocket on PP1 that is formed by residues Asp208_PP1_, Asp210_PP1_, Asn219_PP1_ and Asp220_PP1_ (Fig. 3*F*). This interaction is critical as it optimally positions I3 residues ^60^CCC^62^, that is, the CCC motif, to bind directly over the PP1 active site (Fig. 3, *A* and *F*). Finally, only Cys60 in the CC motif has electron density in the SPI complex (Fig. S3).

### SDS22 weakens the affinity of I3 for PP1 *via* the I3 CCC motif

**Figure 4:**
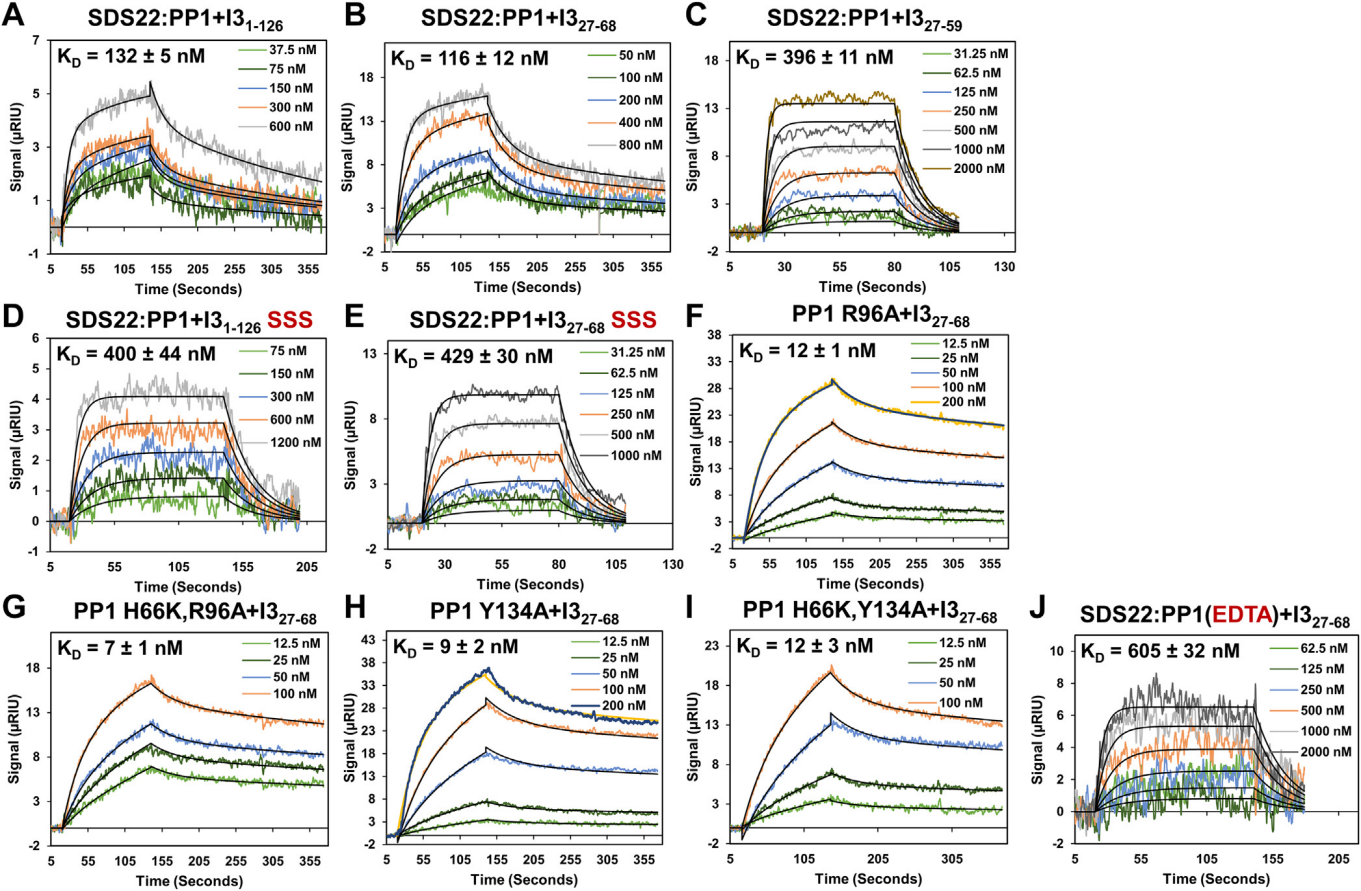
**Role of PP1 active site metal M2 in I3 binding.***A*, SPR sensorgram between I3 and SDS22_56-360_:PP1α_7-330_. *B*, SPR sensorgram between I3_27-68_ and SDS22_56-360_:PP1α_7-330_. *C*, SPR sensorgram between I3_27-59_ and SDS22_56-360_:PP1α_7-330_. *D*, SPR sensorgram between the I3 SSS and SDS22_56-360_:PP1α_7-330_. *E*, SPR sensorgram between I3_27-68_ SSS and SDS22_56-360_:PP1α_7-330_. *F*, SPR sensorgram between I3_27-68_ and PP1α_7-330_ R96A. *G*, SPR sensorgram between I3_27-68_ and PP1α_7-330_ R96A/H66K. *H*, SPR sensorgram between I3_27-68_ and PP1α_7-330_ Y134A. *I*, SPR sensorgram between I3_27-68_ and PP1α_7-330_ Y134A/H66K. *J*, SPR sensorgram between I3_27-68_ and metal-free SDS22_56-360_:PP1α_7-330_ (EDTA treated).

Although the structures of I3 in the I3:PP1 dimer and the SPI trimer complex are largely conserved, the conformations of PP1 between the two structures differ, with PP1 adopting the SDS22-bound conformation in the SPI complex. To determine if this change alters the affinity of I3 for PP1, we used mutagenesis and surface plasmon resonance (SPR). Previously, we showed that I3 (FL and I3_27-68_) binds PP1α_7-330_ with a K_D_ of ∼10 nM and does so with biphasic binding kinetics with the slower phase reflecting the binding of the CCC motif to the PP1 active site (24). Repeating these experiments with I3 (FL and I3_27-68_) and SDS22:PP1 showed that while the biphasic nature of the binding is conserved, the affinities for both I3 constructs for SDS22:PP1 are ∼10-fold weaker than those observed for PP1 alone (FL I3_1-126_ K_D_, 132 ± 5 nM; I3_27-68_ K_D_, 116 ± 12 nM, Figure 4, *A* and *B*, Table 2). To identify the I3 residues that contributed to the reduced affinity, we tested two additional I3 variants, I3_27-59_, which lacks the CCC motif (I3_ΔCCC_), and I3_27-68_ SSS, in which the CCC motif is replaced with SSS. We previously showed that either deleting, I3_ΔCCC_, or mutating, CCC→SSS, the CCC motif weakens the affinity of I3 for PP1 ∼40-fold, highlighting the contribution of the CCC motif for binding PP1 (24). SPR showed I3_ΔCCC_ binds SDS22:PP1 only ∼3.5-fold weaker than I3_27-68_ (I3_ΔCCC_ K_D_, 396 ± 11 nM, Fig. 4*C*, Table 2), with an affinity nearly identical to that measured between I3_ΔCCC_ and PP1 alone (24). This suggests that the 10-fold weaker affinity of I3 for SDS22:PP1 *versus* PP1 alone is due to the interaction of the CCC motif with PP1. To further test this hypothesis, we repeated the SPR measurement with two I3 variants in which the CCC motif is mutated to SSS: I3_1-126_ SSS and I3_27-68_ SSS (Fig. 4, *D* and *E*). As observed with I3_ΔCCC_, the K_D_ values of both variants with SDS22:PP1 are nearly identical to that observed for PP1 alone and similar to that obtained with I3_ΔCCC_. Furthermore, the binding data for the CCC-defective I3 constructs with SDS22:PP1 is best described *via* single exponential global fit, with fast on- and off-rates, *i.e.*, exactly what we observed for I3_ΔCCC_ binding to PP1. Together, these data demonstrate that the 10-fold weaker binding of I3 to SDS22:PP1 *versus* PP1 is strictly due to a weaker interaction at the PP1 active site with the I3 CCC motif.

**Table 2:** I3 binding to the SDS22:PP1 complex

PP1 and I3 variants	Binding		
SDS22:PP1 *versus* I3 variants	K_D_ (nM)	Fit	n
**I3**_**1-126**_			
I3_1-126_	132 ± 5	2	3
SSS: I3_1-126_ CCC→SSS	400 ± 44	1	3
**I3**_**27-68**_			
I3_27-68_	116 ± 12	2	3
I3_27-68_ (PP1 EDTA chelated)	605 ± 32	1	3
SSS: I3_27-68_ CCC→SSS	429 ± 30	1	3
**I3**_**27-68**_**deletion variants**			
CCC_deletion_: I3_27-59_ (Δ60–68)/I3_ΔCCC_	396 ± 11	1	3
**I3**_**27-68**_***versus* different PP1 variants**			
PP1_7-330_ R96A	12 ± 1	2	3
PP1_7-330_ R96A/H66K	7 ± 1	2	3
PP1_7-330_ Y134A	9 ± 2	2	3
PP1_7-330_ Y134A/H66K	12 ± 3	2	3

Bold highlighting different I3 constructs and PP1 variants.

### The weakened affinity of I3 for SDS22:PP1 is due to the M2 metal

The two main differences between PP1 and SDS22:PP1 are the unwinding of loop Tyr134, which results in movements of Tyr134 and Arg96 by 10 Å and 4 Å, respectively, between the two states to facilitate SDS22 binding, and the lack of the PP1 M1 metal (22). To determine if one or both differences contribute to the weakened affinity of I3 for SDS22:PP1, we first generated PP1 variants in which Tyr134 and Arg96 were mutated to Ala (PP1_Y134A_, PP1_R96A_) and measured their affinities for I3. We previously showed that PP1_Y134A_ fails to bind SDS22 while PP1_R96_ stabilizes the SDS22-bound conformation by moving out of the active site (22)) and measuring their affinities (Fig. 4, *F* and *I*). No statistically meaningful changes in the binding of I3 to these PP1 variants were measured, highlighting that although these PP1 residues are important for SDS22 binding to PP1, their conformational change does not influence the interaction between I3 and the M2 metal in the SDS22:PP1 complex.

We then tested the role of the M2 metal. We previously showed that the affinity of I3 for PP1 does not depend on the presence of the M1 metal because I3 binds both PP1 and PP1 H66K (a stable PP1 variant that lacks the M1 metal; (22)) with identical affinities (24). Our observation that I3 binds SDS22:PP1 10-fold more weakly than PP1 alone, coupled with our previous data that strongly points to a role for the M2 metal in I3 binding, strongly suggests that the SDS22:PP1 complex likely exists in both an M2-bound and M2-free state; *i.e.*, that the PP1 M2 metal exchanges more readily in the SDS22:PP1 complex than PP1 alone. The absence of an M2 metal in a subpopulation of SDS22:PP1 complexes would result in an overall weaker interaction of SDS22:PP1 with I3. To test this, we generated PP1 variants unable to bind the M2 metal; however, these proteins failed to fold and purify, re-confirming that free PP1 rapidly unfolds in the absence of the M2 metal. We then incubated SDS22:PP1 with EDTA to extract the M2 metal and repeated the SPR experiment; SDS22 stabilizes metal-free PP1, as determined using thermal denaturation assays. SDS22:metal-free PP1 remains folded with a T_m_ = 62 °C *versus* T_m_ = 64 °C for untreated SDS22:PP1. Consistent with our hypothesis, I3 binds SDS22:PP1_M2-metal-free_ even more weakly than SDS22:PP1, with fast on- and off-rates best described by a single exponential, *i.e.*, in a manner identical to I3 variants lacking the CCC motif (Fig. 4*J*).

## Discussion

**Figure 5:**
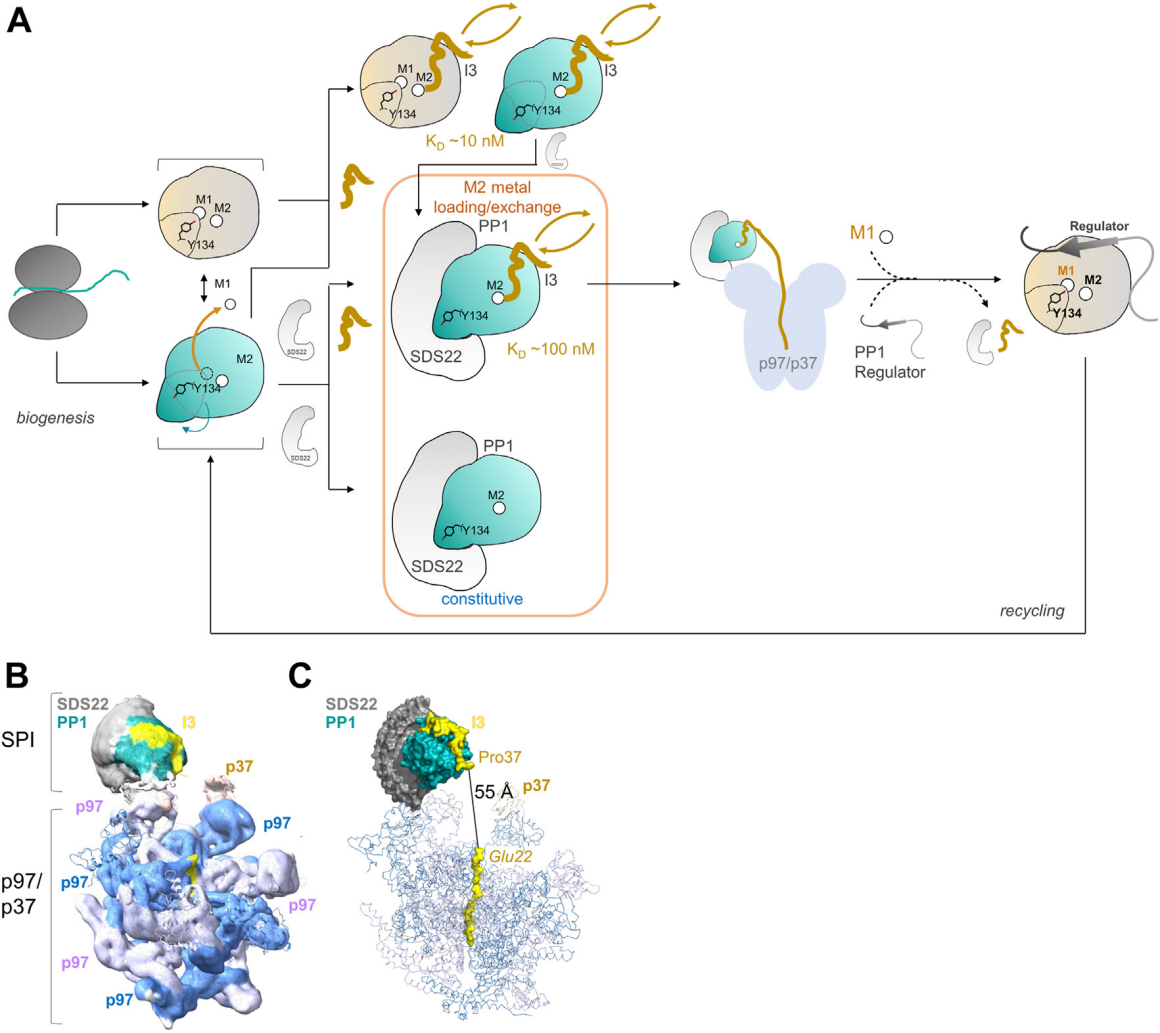
**Model for PP1 holoenzyme assembly and the effect of SDS22 on the I3:PP1 complex.***A*, PP1, either newly synthesized or recycled from previous holoenzyme complexes, is present as either a metal-loaded (beige) or an M1 metal-deficient conformation (*teal*). I3 (*dark yellow*) readily associates and dissociates (indicated by the *double arrows*) with either metal-loaded or M1 metal-free PP1 with binding affinities of ∼10 nM. In contrast, SDS22 selectively binds M1 metal-free PP1 and this binding is constitutive in the absence of other factors. The binding of I3 to SDS22:M1 metal-free PP1 complexes is 10-fold weaker than to PP1 alone (metal-loaded or M1 metal-free), due to a change in M2 metal affinity, suggesting that in addition to serving as a PP1-storage protein, SDS22 also facilitates M2 metal-loading and/or exchange. I3-mediated recruitment of the p97/p37 complex (*light blue*, p37 not shown for clarity) results in the dissociation of SDS22 (and I3) from PP1 and the association of the M1 metal and regulator for the formation of an active PP1 holoenzyme. *B*, the SPI complex was docked into the SPI-p97/p37 cryo-EM map (EMD-15861) using ChimeraX; p97 subunits are dark and light blue, p37 is in beige, SDS22 in grey, PP1 in teal and I3 in yellow. *C*, same as *B*, except with the optimally docked SPI coordinates replacing those of SDS22 and PP1 in the SPI PDB file (PDBID 8B5R). The distance between the N-terminal residue in the SPI crystal structure (Pro37) and the topmost residue of the substrate present in the p97 substrate channel (attributed to I3, also *yellow*) is illustrated by a *black line* and labeled.

The two most ancient interactors of PP1 are SDS22 and I3 (23). Early work established that while both regulators form dimeric, inhibitory complexes with PP1, they also assemble into a stable, trimeric inhibitory complex, SPI. The triple complex formation has only been observed for a handful of PP1 regulators, namely spinophilin and inhibitor-2 (spinophilin:PP1, I2:PP1 and spinophilin:PP1:I2 (38)) and GADD34 and inhibitor-1 (GADD34:PP1, I1:PP1, GADD34:PP1:I1 (39)). However, unlike the spinophilin:PP1:I2 and GADD34:PP1:I1 complexes, in which spinophilin/I2 and GADD34/I1 complex compete for specific PP1 SLiM binding pockets (33, 40, 41), SDS22 and I3 in SPI bind PP1 *via* completely distinct interfaces. This explains why SPI complex formation does not alter the interactions observed in the two dimeric complexes; *i.e.*, the structures of SDS22 and I3 bound to PP1 are essentially identical between the SPI and the individual SDS22:PP1 and I3:PP1 complexes. The key exception is that the conformation of PP1 in SPI is identical to that observed in the SDS22:PP1 complex, where PP1 lacks its M1 metal and the Tyr134 loop is unwound, positioning Tyr134 to bind SDS22 and not the PP1 active site pocket. While overlays of the SPI complex with the SDS22:PP1 and I3:PP1 complex reveal no change in the overall structure of I3, SPR measurements highlighted that I3 binds 10-fold weaker to the SDS22:PP1 complex than to PP1 alone. We showed that the change in affinity is solely due to a change in the I3 CCC motif binding to the PP1 M2 metal. Together, these data are consistent with a model in which SDS22 binding to PP1 not only stabilizes PP1 in a M1-metal-free conformation but, simultaneously, weakens its affinity for the M2 metal (Fig. 5*A*). Although it is still not known how metals are loaded into PP1 (or the entire PPP family), these data suggest that, in addition to maintaining a pool of inactive PP1 poised for PP1 holoenzyme assembly when needed by the cell, SDS22 also facilitates PP1 metal exchange. Indeed, the ability of SDS22 to constitutively bind PP1 provides a unique platform to stabilize the PP1 fold, while allowing for insertion or exchange of the M2 metal (PP1 variants unable to bind the M2 metal fail to fold, with M2 metal loss also associated with PP1 unfolding (24)). Our data do not identify structural changes, indicating that they might not involve changes in the conformation of PP1 but instead that SDS22 alters the PP1 active site dynamics, facilitating M2 exchange. The role that I3 may play in PP1 metal exchange, potentially *via* its CCC motif, is a major outstanding question in the field.

An additional key function of I3 is to recruit the p97-p37 disaggregase, a AAA+ ATPase to SPI trimers to facilitate PP1 holoenzyme formation (28, 31, 42). Current data are consistent with a model in which the energy of p97-mediated ATP hydrolysis dissociates SDS22 from PP1 (22, 31). This is because while I3 readily associates and dissociates from SDS22:PP1 complexes in the absence of p97/p37, SDS22 does not; instead, once SDS22 is bound to M1-metal free PP1, it does not dissociate without I3, p97/p37 and ATP (22) (Fig. 5*A*). This model is consistent with the most recent 6.1 Å resolution cryo-EM structural data of the SPI-p97-p37 complex, in which a peptide, likely I3, is bound in the p97 hexamer channel and SDS22 from the SPI complex binds directly to p97 (31). Because only the SDS22:PP1 complex coordinates were available at the time of publication, unaccounted-for density was hypothesized to correspond to I3. We confirmed this by docking the SPI complex into the SPI-p97-p37 complex cryo-EM map (EMD-15861 (31))(Fig. 5*B*). The unaccounted-for density is located exactly where I3 residues 37 to 60 bind, *i.e.*, from the RVxF binding pocket to the acidic groove to the PP1 catalytic site. Furthermore, the distance between I3 residue Pro37 and the residue modeled into the topmost density of the p97 substrate channel is 52 Å. If, as hypothesized, this channel density corresponds to I3, the topmost residue of the channel peptide corresponds to I3 Glu22; this distance is compatible with the missing intervening 15 amino acids from I3 being in a fully extended conformation. How the energy from ATP hydrolysis is transferred from I3 to the SDS22:PP1 interaction remains to be elucidated. Furthermore, if and how I3, *via* its CCC motif, participates in PP1 metal loading is also under active investigation. It is established that PP1 variants unable to bind the M1 metal (H66K) do not dissociate from SDS22 in cells (22), demonstrating that metal loading is a key step in PP1 holoenzyme formation.

## Experimental procedures

### Bacterial protein expression

The coding sequence of human I3 was synthesized by DNA 2.0. The coding sequences of human I3_1-126_ and I3_27-68_ were subcloned into a pET-M30-MBP vector containing an N-terminal his_6_-tag followed by maltose binding protein (MBP) and a TEV protease cleavage site. *E. coli* BL21 (DE3) cells (Agilent) were transformed with I3 expression vectors. Freshly transformed cells were grown at 37 °C in LB broth containing kanamycin antibiotics (50 μg/ml) until they reached an optical density (OD_600_) of 0.6 to 0.8. Protein expression was induced by the addition of 1 mM β-D-thiogalactopyranoside (IPTG) to the culture medium, and cultures were incubated with shaking overnight (18–20 h) at 18 °C. Cells were harvested by centrifugation (6000*g*, 15 min, 4 °C) and stored at −80 °C until purification. I3 variant I3_27-59_ was generated by the introduction of a stop codon after the respective site; I3_1-126_ C60S/C61S/C62S, and I3_27-68_ C60S/C61S/C62S were generated by site-directed mutagenesis. All variants were sequence-verified and expressed as described above. Cloning and expression of PP1α_1-330_, PP1α_7-330_, PP1α_7-330_ H66K, PP1α_7-330_ R96A, PP1α_7-330_ R96A/H66K, PP1α_7-330_ Y134A, and PP1α_7-330_ Y134A/H66K was performed as previously described (32, 34).

### Protein purification

Cell pellets expressing I3 (I3_1-126_, I3_27-68_, I3_27-59_, I3_1-126_ C60S/C61S/C62S, I3_27-68_ C60S/C61S/C62S) were resuspended in ice-cold lysis buffer (50 mM Tris pH 8.0, 500 mM NaCl, 5 mM imidazole, 0.1% Triton X-100 and a protease inhibitor tablet [ThermoFisher Scientific]) and lysed by high pressure homogenization (Avestin EmulsiFlex C3). Lysate was clarified by centrifugation (45,000*g*, 45 min, 4 °C), and the supernatant was loaded under gravity onto Ni^2+^-NTA beads (GE Healthcare) pre-equilibrated with 50 mM Tris pH 8.0, 500 mM NaCl and 5 mM imidazole. Protein was eluted in four column volumes (20 ml) using 50 mM Tris pH 8.0, 500 mM NaCl and 500 mM imidazole. Fractions with I3 were pooled and dialyzed with TEV protease overnight at 4 °C against 50 mM Tris pH 8.0, 500 mM NaCl, and 20 mM DTT to cleave the MBP-His_6_-tag. The cleaved protein was heat purified at 95 °C (15 min), filtered using a 0.22 μm filter (Millipore), concentrated and further purified using size-exclusion chromatography (SEC; Superdex 75 26/60 [Cytiva]) equilibrated in either NMR Buffer (20 mM Bis-Tris pH 6.8, 500 mM NaCl, 0.5 mM TCEP) or SEC/SPR buffer (20 mM Tris pH 8.0, 500 mM NaCl, 0.5 mM TCEP and 1 mM MnCl_2_). SEC fractions were pooled, concentrated, and stored at −20 °C.

PP1 was purified as described previously (32). Briefly, PP1 (PP1α_1-330_, PP1α_7-330_, PP1α_7-330_ H66K, PP1α_7-330_ R96A, PP1α_7-330_ R96A/H66K, PP1α_7-330_ Y134A and PP1α_7-330_ Y134A/H66K) was lysed in PP1 Lysis Buffer (25 mM Tris pH 8.0, 700 mM NaCl, 5 mM imidazole, 1 mM MnCl_2_, 0.1% Triton X-100), clarified by centrifugation (45,000*g*, 45 min, 4 °C) and immobilized on Ni^2+^-NTA resin. Bound His_6_-PP1 was washed with PP1 Buffer A (25 mM Tris pH 8.0, 700 mM NaCl, 5 mM imidazole, 1 mM MnCl_2_), followed by a stringent wash containing 6% PP1 Buffer B (25 mM Tris pH 8.0, 700 mM NaCl, 250 mM imidazole, 1 mM MnCl_2_) at 4 °C. The protein was eluted using PP1 Buffer B and His_6_-tagged PP1 was purified using SEC (Superdex 200 26/60 [Cytiva]) pre-equilibrated in SEC Buffer (20 mM Tris pH 8, 500 mM NaCl, 0.5 mM TCEP, 1 mM MnCl_2_). Peak fractions were incubated overnight with TEV protease at 4 °C. The cleaved protein was incubated with Ni^2+^-NTA beads (GE Healthcare), the flow-through was collected and immediately used. All experiments were performed with freshly purified PP1.

### Cloning and mammalian protein expression

PP1α_7-300_ and SDS22_56-360_ were cloned into pcDNA3.4_K_RP1B as previously described (43). I3_27-68_ was cloned into modified pcDNA3.4_K_RP1B vector pCDNA3.4_K_GFP_RP1B with N-terminal His_6_-GFP-tag and a TEV cleavage site. The plasmids were amplified and purified using the NucleoBond Xtra Maxi Plus EF (Macherey-Nagel). Transfection was carried out using 500 ml culture medium (SMM 293-TII, Sino Biological Inc) in 2 L flasks (ThermoFisher Scientific) according to the manufacturer’s protocol in an incubator at 37 °C and 8% CO_2_ under shaking (125 rpm). Prior to transfection, the cell density was adjusted to 2.5 × 10^6^ cells/ml using fresh medium. Equal ratio of plasmid DNA of PP1_7-300_, SDS22_56-360,_ and I3_27-68_ (total DNA per mL of the final culture medium = 1.0 μg) were diluted into 25 ml of Opti-MEM reduced serum medium (ThermoFisher Scientific). In a separate tube, 3× the amount of PEI (Polysciences), compared to DNA, was diluted into the same volume of Opti-MEM medium (ThermoFisher Scientific). The SDS22_56-360_ only expression was carried out with 1 μg/ml DNA in 25 ml Opti-MEM. The DNA and PEI mixtures were combined and incubated for 10 min at RT, before being added into the cell culture. Valproic acid (2.2 mM final concentration, Sigma) was added to the cells 4 h after transfection. The cells were harvested 72 h after transfection by centrifugation (2000*g*, 20 min, 4 °C). All protein pellets were stored at −80 °C.

### SPI complex purification

Cell pellet was resuspended in ice-cold Lysis Buffer (50 mM Tris pH 8.0, 500 mM NaCl, 5 mM imidazole, 0.1% Triton X-100) supplemented with an EDTA-free protease inhibitor tablet (ThermoFisher Scientific) and lysed using high-pressure homogenization (EmulsiFlex C3, Avestin). The lysate was clarified by centrifugation (42,000*g* for 45 min at 4 °C) and filtered (Millex-GP 0.22 μm PES syringe filter [Millipore]) before loading onto a pre-equilibrated 5 ml HisTrap Fast Flow column (Cytiva) equilibrated in Buffer A (50 mM Tris pH 8.0, 500 mM NaCl, 5 mM imidazole) using a BioRad NGC chromatography system at 4 °C. Before protein elution, the column was washed with 6% Buffer B and then 40 ml of 6% His Buffer B containing 5 mM ATP-Mg. The SPI complex was eluted using a 6 to 65% Buffer B (50 mM Tris pH 8.0, 500 mM NaCl, 500 mM imidazole) gradient. Fractions containing the SPI complex were pooled and dialyzed in a Dialysis Buffer (50 mM Tris pH 8.0, 250 mM NaCl, 0.5 mM TCEP) with TEV protease added. The next day, subtraction purification was performed using Ni-NTA beads (Prometheus, Genesee Scientific), and the flow through fractions that contained the SPI complex were collected. The SPI complex was further purified using SEC (Superdex75 26/60 [Cytiva]) in SEC buffer (20 mM Tris pH 8.0, 250 mM NaCl, 1 mM TCEP).

Purification of SDS22_56-360_ for SPR was achieved as follows. An SDS22_56-360_ cell pellet was resuspended in ice-cold lysis buffer (25 mM Tris pH 8.0, 0.5 M NaCl, 5 mM imidazole, 0.1% Triton X-100) with an EDTA-free protease-inhibitor cocktail tablet (ThermoFisher Scientific). The resuspended cells were lysed using an Emulsiflex 3C homogenizer (Avestin). The cell lysate was clarified by centrifugation at 40,000*g* for 45 min and the resulting supernatant was filtered using a 0.22 mm syringe filter (Millipore). The filtered supernatant was loaded onto a HisTrap HP column (Cytiva) pre-equilibrated with buffer A (25 mM Tris pH 8.0, 500 mM NaCl, 5 mM imidazole). The column was washed with buffer A until the baseline was reached, followed by a wash with 8% buffer B (25 mM Tris pH 8.0, 500 mM NaCl, 250 mM imidazole); after this, the protein was eluted using a linear gradient of 8 to 60% buffer B. The fractions containing SDS22 were pooled, and the His_6_-tag was cleaved using TEV (overnight incubation at 4 °C in SDS22 buffer: 20 mM Tris pH 8.0, 500 mM NaCl, 0.5 mM TCEP). The cleaved His_6_-tag and TEV were removed *via* subtraction purification.

### Crystallization

SDS22_56-360_:PP1α_7-300_:I3_27-68_ (SPI) was concentrated to 8.5 mg/ml. Crystallization was performed using Gryphon LCP (Art Robbins) into Intelli 3-well plate (Art Robbins). Small cubic-shaped crystals were identified in two conditions (0.1 M HEPES pH 7.5, 1.4 M sodium citrate and 1.8 M sodium phosphate monobasic monohydrate, potassium phosphate dibasic pH 8.2). Following cryoprotection, the crystals diffracted to low (>10 Å) resolution. Crystals were then transferred to cryoprotectant solution (1.8 M sodium phosphate monobasic monohydrate, potassium dibasic pH 8.2 supplemented with 25% glycerol) and the drop was allowed to evaporate overnight to facilitate crystal dehydration. Crystal diffraction improved significantly, ultimately resulting in a dataset processed to 3.2 Å.

### Structure determination

The structure of the SPI complex was determined by molecular replacement using Phaser as implemented in PHENIX (44); the crystal structure of the SDS22:PP1 complex (PDBID 6OBN) was used as the search model. A solution was obtained in space group F 4 3 2. The model was completed using iterative rounds of refinement in PHENIX and manual building in WinCoot 0.9.8 (45).

### NMR spectroscopy

NMR experiments were acquired on a Bruker Neo 600 MHz spectrometer, equipped with a TCI HCN-z cryoprobe at 283 K. NMR buffer was 20 mM Bis-Tris pH 6.8, 500 mM NaCl, 0.5 mM TCEP and 90% H_2_O/10% D_2_O. The interaction between I3_1-126_ with SDS22_56-360_:PP1α_1-330_ was studied by direct comparison of the 2D [^1^H,^15^N] HSQC spectra of free ^15^N-labeled I3_1-126_ and in complex with PP1α_1-330_. The spectra were processed using Topspin 4.1 and analyzed using NMRFAM-Sparky (46).

### Extraction of M2 from SDS22:PP1

3 mM EDTA (100:1 ratio of EDTA *versus* SDS22:PP1 complex) in Exchange Buffer (20 mM Tris pH 8.0, 250 mM NaCl, 0.5 mM TCEP) was added to freshly purified SDS22:PP1 complex and the complex was dialyzed 3-times against fresh EDTA containing Exchange Buffer for 12 h at 4 °C. The complex was then dialyzed for 12 h at 4 °C against Exchange Buffer to remove EDTA. Melting temperature (T_m_) measurements were performed on a Tycho NT.6 (Nanotemper) using standard capillaries (10 μl) and a 30 °C/min ramp (from 35 to 95 °C). The data were evaluated using the Tycho NT.6 software version 1.1.5.668.

### Surface plasmon resonance

SPR measurements were performed using a 4-channel Reichert 4SPR instrument fitted with an autosampler and a degassing pump (Reichert Technologies). SPR buffers containing 20 mM Tris pH 8.0, 500 mM NaCl, 0.5 mM TCEP, 0.05% Tween-20 were prepared, sterile filtered, and degassed in autoclaved glassware prior to each experiment. Running buffer was used to prime and run both the sample and syringe pump reservoirs. Gold sensorchips modified with Ni-NTA-functionalized polycarboxylate (NiHC200M; XanTec Bioanalytics GmbH) were installed and equilibrated under flow conditions (100 μl/min) for ≥60 min at 25 °C. Surface contaminants were cleared from the chip surface by a pair of 120 μl injections of 2 M NaCl and 10 mM NaOH during the equilibration step. Experiments were conducted at 25 °C with a 5 Hz sampling rate and were initiated by injecting 180 μl of His_6_-PP1 (PP1α_7-330_, PP1α_7-330_ R96A, PP1α_7-330_ R96A/H66K, PP1α_7-330_ Y134A and PP1α_7-330_ Y134A/H66K) constructs or SDS22_56-360_:PP1α_7-330_ (40–80 nM) diluted in 20 mM Tris pH 8.0, 500 mM NaCl, 0.5 mM TCEP, 0.05% Tween-20 onto channels 1, two and three for 180 s at 50 μl/min which resulted in between 200 to 450 μRIU of surface loading (channel four was used as reference). The sensorchip was allowed to equilibrate for 5 min at 50 μl/min prior to initiation of experiments. The concentrations of I3_1-126_ and its variants (I3_1-126_ C60S/C61S/C62S, I3_27-68_, I3_27-59,_ and I3_27-68_ C60S/C61S/C62S) were measured using AccuOrange Protein Quantification Kit (Biotium). For measurements, I3_1-126_ and its variants were diluted into running buffer from concentrated stocks, and a series of injections at different I3 concentrations were applied. 60 to 120 μl samples of I3 were respectively injected for 60 to 120 s at 50 μl/min followed by a dissociation step of 120 s to 300 s. For all experiments, buffer blank injections were recorded before and after sample injections to achieve double referencing. Technical replicates were obtained by utilizing three channels per chip coupled with stripping of the sensorchip with 350 mM EDTA pH 8, reconditioning the surface with 10 mM NaOH to remove non-specifically bound PP1 aggregates, charging the surface with 40 mM NiSO_4_, and reloading fresh PP1 onto the surface. All replicates were generated with freshly diluted PP1 and I3. Kinetic parameters were determined by curve-fitting using TraceDrawer software (Ridgeview Instruments AB) fit with a one-to-one one-state or one-to-one two-state model. Statistical analyses of SPR data were performed using Microsoft Excel.

## Data availability

The atomic coordinates and structure factors generated in this study have been deposited in the PDB database under accession code 8U5G (https://doi.org/10.2210/pdb8U5G). All NMR intensity and SPR data generated in this study are available at Figshare (10.6084/m9.figshare.24279511).

## Supporting information

This article contains supporting information.

## Conflict of interest

The authors declare no competing interests. The funders had no role in study design, data collection and analysis, decision to publish, or preparation of the manuscript.
