# Supplementary material for: The SDS22:PP1:I3 complex: SDS22 binding to PP1 loosens the active site metal to prime metal exchange
Source: J Biol Chem. 2023 Nov 30;300(1):105515. doi: 10.1016/j.jbc.2023.105515 (PMC10776994; doi:10.1016/j.jbc.2023.105515)
Supplement: Supporting Figures S1–S3 [file mmc1.pdf]

Supplemental Information for

The SDS22:PP1:I3 complex: SDS22 binding to PP1 loosens active site metal binding to prime metal exchange

Meng S. Choy[1], Gautam Srivastava[1], Lucy C. Robinson[2], Kelly Tatchell[2], Rebecca Page[3,*] & Wolfgang Peti[1,*]

[1]Department of Molecular Biology and Biophysics, University of Connecticut Health Center, Farmington, CT 06030; [2]Department of Biochemistry and Molecular Biology, Louisiana State University Health Sciences Center, Shreveport, LA 71130; [3]Department of Cell Biology, University of Connecticut Health Center, Farmington, CT 06030

*To whom correspondence should be addressed. Rebecca Page: rpage@uchc.edu; Wolfgang Peti: e-mail: peti@uchc.edu

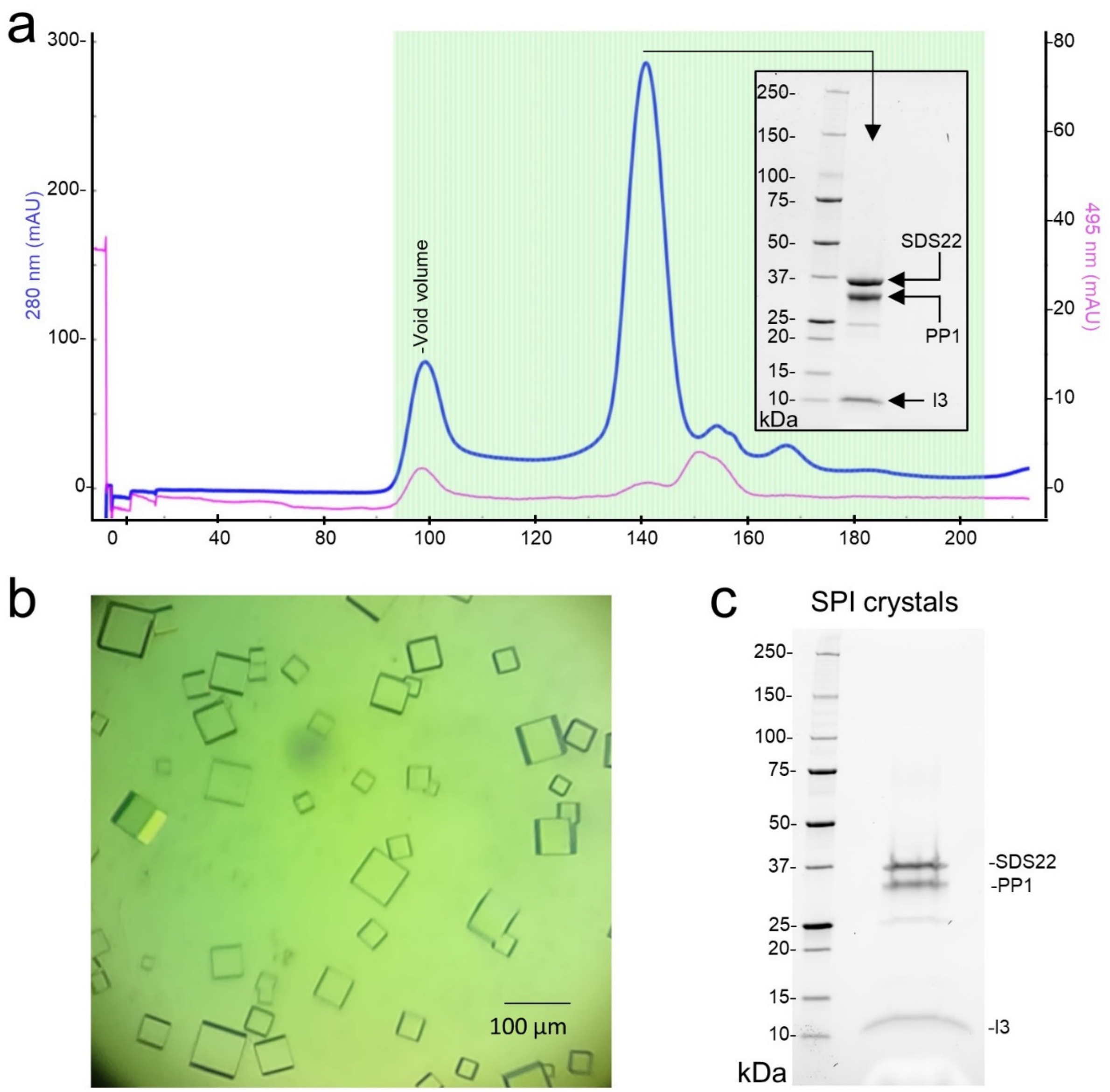

Supplemental Figure S1. Purification and crystallization of the SPI complex. a. SEC chromatogram of purified SPI complex expressed in Expi293F cells; *inset,* stain-free SDS-PAGE gel of the peak pooled for crystal formation. **b**. typical crystals of the SPI complex. **c**. Stain-free SDS-PAGE gel SPI crystals (washed in crystallization buffer 3x prior to incubation with SDS-loading buffer).

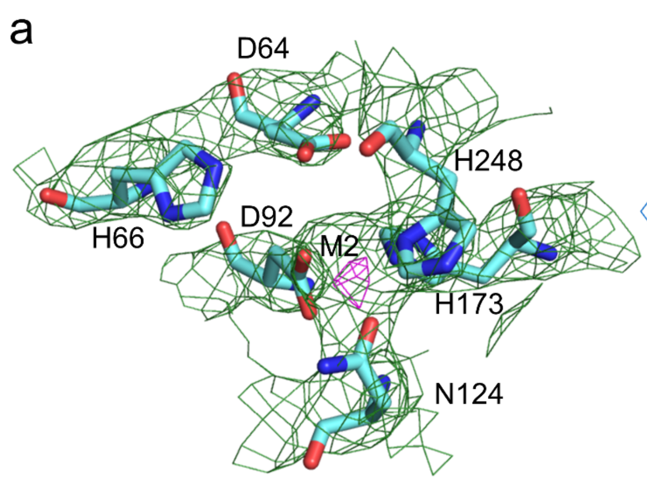

Supplemental Figure S2. The M2 metal at the PP1 active site in the SPI complex. **a.** Omit map (green mesh) and anomalous map (magenta mesh), both at sigma level = 3.0, confirmed the presence of a single metal at the M2 metal site of the SPI complex. **b.** 2Fo-Fc map (blue mesh) at sigma level 1.0 after refinement with iron (Fe) at the M2 site (Orange sphere = Fe, occupancy = 0.92). Fe is coordinated by Asp92, Asn124, His173 and His248 of PP1, shown here with black dash lines.

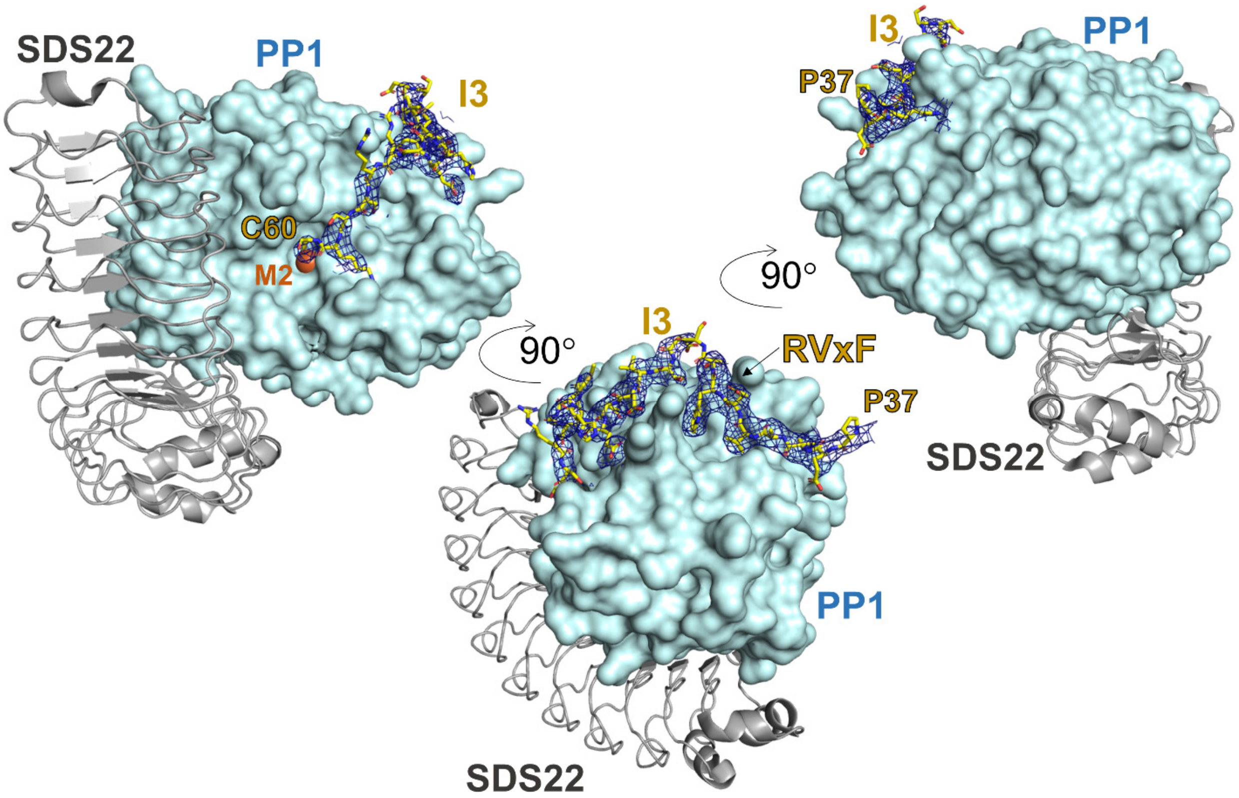

Supplemental Figure S3. Electron density of I3 in the SPI complex. Omit map of I3 at sigma = 1.0 (blue mesh). I3 yellow stick; PP1 cyan surface; SDS22 grey cartoon.
